# Inflammation-Related MicroRNAs Are Associated with Plaque Stability Calculated by IVUS in Coronary Heart Disease Patients

**DOI:** 10.1155/2019/9723129

**Published:** 2019-12-01

**Authors:** Guo-fu Zhu, Tianshu Chu, Zhimin Ruan, Mingguo Zhang, Mingli Zhou, Qian Zhang, Ran Zhang, Liyong Wu

**Affiliations:** Department of Cardiology, The Second Affiliated Hospital of Kunming Medical University, Kunming, Yunnan 650101, China

## Abstract

**Objectives:**

This study aimed to investigate the association between inflammation-related microRNAs (miR-21, 146a, 155) and the plaque stability in coronary artery disease patients.

**Methods:**

The expression of miR-21, 146a, and 155 was measured by real-time PCR in 310 consecutive patients. The level of hs-CRP, IL-6, and IL-8 was measured by ELISA. The plaque stability of coronary stenotic lesions was evaluated with intravascular ultrasound (IVUS).

**Results:**

(1) The levels of hs-CRP, IL-6, and IL-8 were significantly increased in the UAP and AMI groups compared with the CPS group (*P* < 0.01). (2) The expression of miR-21 and miR-146a in peripheral blood mononuclear cells (PBMCs) and plasma was significantly higher in CAD patients compared with non-CAD patients, whereas the miR-155 expression in PBMCs and plasma was significantly lower in patients with CAD. (3) The miR-21 expression in PBMCs was higher in UAP and AMI groups compared with CPS group. The miR-146a expression in PBMCs was higher in SAP, UAP, and AMI groups than in CPS group. Although the level of miR-155 in PBMCs was lower in SAP, UAP, and AMI groups than in CPS group. The expression patterns of miR-21, miR-146a, and miR-155 in plasma were consistent with those of PBMCs. (4) The expressions of miR-21 and miR-146a in PBMCs and plasma were significantly higher in the vulnerable plaque group than those in stable plaque group. While miR-155 in PBMCs and plasma was significantly lower in vulnerable plaque group compared with stable plaque group. (5) The levels of miR-21 and miR-146a in PBMCs and plasma were significantly higher in soft plaque group than in fibrous plaque group and calcified plaque group. However, miR-155 in PBMCs and plasma was significantly lower in soft plaque group.

**Conclusions:**

The expression of miR-21 and miR-146a are associated with the plaque stability in coronary stenotic lesions, whereas miR-155 expression is inversely associated with the plaque stability.

## 1. Introduction

At present, cardiovascular disease is the leading cause of death throughout the world. The mechanism of coronary artery disease is atherosclerosis (AS), which leads to coronary artery stenosis and myocardial ischemia. Atherosclerotic disease is a chronic inflammatory disease characterized by the accumulation of inflammatory cells in the vessel wall. Accumulating evidence indicates that inflammation plays a pivotal role in AS [1–3]. MicroRNAs have been demonstrated to be associated with inflammation and cardiovascular disease [4–7]. MicroRNAs (MiR) are small noncoding RNA that posttranscriptionally regulate gene expression in 30% of all human genes [8].

The alternation in the expression of microRNAs in some inflammatory diseases has been investigated. For example, both miR-21 and miR-146a were upregulated in psoriasis [9], whereas miR-146a and miR-155 were upregulated in rheumatoid arthritis [10]. MiR-21, miR-146a, and miR-155 were considered to be inflammation-related microRNAs and are associated with coronary artery disease [11]. Our previous studies has also confirmed that the expression of miR-155 in peripheral blood mononuclear cells (PBMCs) and plasma was decreased in patients with coronary heart disease, and it was negatively correlated with the severity of the disease and coronary artery lesions assessed by Gensini score [12]. But whether the miR-21, miR-146a, and miR-155 are associated with plaque stability is still unknown.

Based on the relationships between microRNAs, inflammation, and atherosclerosis, we hypothesized that inflammation-related miRNAs, like miR-21, miR-146a, and miR-155, might play a role in atherosclerosis and plaque stability. The aim of this study is to detect the level of miR-21, miR-146a, and miR-155 in patients with acute myocardial infarction (AMI), unstable angina pectoris (UAP), and stable angina pectoris (SAP) and compare their level in patients with chest pain syndrome (CPS) and to determine whether miR-21, miR-146a, and miR-155 are associated with plaque stability, and the stability of plaque will be evaluated by intravascular ultrasound (IVUS).

## 2. Materials and Methods

### 2.1. Study Population

The study is based on the 1975 Declaration of Helsinki Principles. The protocol was reviewed and approved by the Medical Ethics Committee of the Second Affiliated Hospital of Kunming Medical University (Kunming, China). All the recruited patients were informed about the study, and the written informed consent was obtained from all participants.

All 310 subjects enrolled in this study, including patients with AMI (*n* = 42), UAP (*n* = 97), SAP (*n* = 88), and CPS (*n* = 83), are patients of the Cardiology Department of the Second Affiliated Hospital of Kunming Medical University (Yunnan province, China) from October 2016 to March 2018. The AMI group was composed of 28 men and 14 women with a mean age of 62 ± 7 years. The UAP group was composed of 65 men and 32 women with a mean age of 63 ± 7 years. The SAP group was composed of 52 men and 36 women with a mean age of 62 ± 8 years. The CPS group was composed of 50 men and 33 women with a mean age of 60 ± 8 years. The inclusion and exclusion criteria of AMI, UAP, SAP, and CPS were similar to our previous articles [12]. All patients underwent coronary angiography that was performed via transradial or transfemoral approach. CAD was diagnosed by coronary angiography showing stenosis of one or more coronary arteries with lumen narrowing ≥50% in coronary arteries larger than 1.5 mm in diameter [13]. All coronary angiogram was performed by experienced investigators who were blinded to the study.

### 2.2. IVUS

IVUS was carried out to evaluate the plaque stability of coronary stenotic lesions after coronary angiography. All examinations were done using a iLab system (Boston Scientific, Natick, Massachusetts, USA). IVUS measurements were taken at the sections with minimum lumen area. The vessel and lumen area were traced and measured using planimetry software (Echoplaque2, INDEC Systems, Mountain View, California). Lesion plaque composition was evaluated visually according to the American College of Cardiology Clinical Expert Consensus, about its Acquisition, Measurement, and Reporting of IVUS Studies [14]. Plaque with ultrasound attenuation was determined as backward signal attenuation behind a plaque without dense calcium. A lipid pool-like image was determined as echolucent material covered with a high-echoic layer or a pooling of low-echoic material. The diagnostic criteria of vulnerable plaque was as follows: (1) fiber cap thickness ≤0.7 mm; (2) lipid core area ≥1.0 mm^2^; (3) ratio of lipid core area to plaque area ≥20%; (4) eccentric patch; and (5) the presence of >180° of ultrasound attenuation without dense calcium [15]. According to the nature of the plaque, patients were divided into 3 groups: calcified plaque group, fibrous plaque group, and soft plaque group.

### 2.3. Sample Preparation

Blood samples (5 ml) were collected and transferred to sodium heparin vacutainers (Becton Dickinson, Cedex, France). Plasma were collected after centrifugation (15 min at 1000*g*), and then, PBMCs were isolated by Ficoll-Hypaque density gradient centrifugation within 1 hour.

### 2.4. RNA Isolation and Real-Time PCR

According to the manufacturer's protocols, total RNA was extracted from plasma by TRIzol LS (Invitrogen, Carlsbad, CA, USA), and total RNA was extracted from PBMCs by TriPure (Roche, IN, USA). RNA concentration and purity were measured by a spectrophotometer, and samples were used only if A_260/280_ was between 1.8 and 2.0. Quantitative RT-PCR for miR-21, miR-146a, and miR-155 was performed using SYBR Green PCR kit (Toyobo, Osaka, Japan). In brief, 500 ng total RNA was reverse-transcribed into cDNA. PCR was performed using 2 *μ*l reverse-transcribed samples in a 25 *μ*l of final reaction mixture. The primers specific for hsa-miR-21, miR-146a, and miR-155 (miR-21, miR-146a, and miR-155 specific stem-loop RT primers (RiboBio, GuangZhou, China)) were used according to the manufacturer's instructions. The mRNA levels of miR-21, miR-146a, and miR-155 were normalized to that of *C. elegans* miR-39 control. All RT reactions were run in triplicate using a Bio-Rad IQ5 Detection System. The relative amounts of miR-21, miR-146a, and miR-155 were calculated using the comparative Ct (2^−∆∆CT^) method [16].

### 2.5. Statistical Analysis

Continuous variables were expressed as means ± SD. A Student's *t* test was used to compare the CAD group and non-CAD group. One-way ANOVA was used to determine the overall differences between different independent groups. A two-tailed value of *P* < 0.05 was considered significant. All analyses were performed using GraphPad Prism 5.0 software (GraphPad Software, San Diego, CA).

## 3. Results

### 3.1. Basic Clinical Characteristics of Patients

There were no significant differences in gender, age, and risk factors (which include hypertension, diabetes, hyperlipidemia, and tobacco) among patients with CPS, SAP, UAP, and AMI (Table 1). The levels of hs-CRP (*P*=0.004), IL-6 (*P*=0.002), and IL-8 (*P*=0.002) were significantly increased in the AMI groups compared with the CPS groups. The levels of hs-CRP (*P*=0.005), IL-6 (*P*=0.004), and IL-8 (*P*=0.008) were significantly increased in the UAP groups compared with the CPS groups. In addition, the levels of hs-CRP (*P*=0.018), IL-6 (*P*=0.022), and IL-8 (*P*=0.031) were significantly increased in the AMI groups compared with the UAP groups. However, there was no significant difference in level of hs-CRP and IL-6 between CPS group and SAP group. There was no significant difference in level of hs-CRP and IL-8 between UAP group and SAP group.

### 3.2. The Relationship between the Expression of miR-21, miR-146a, and miR-155 and CAD

**Figure 1:**
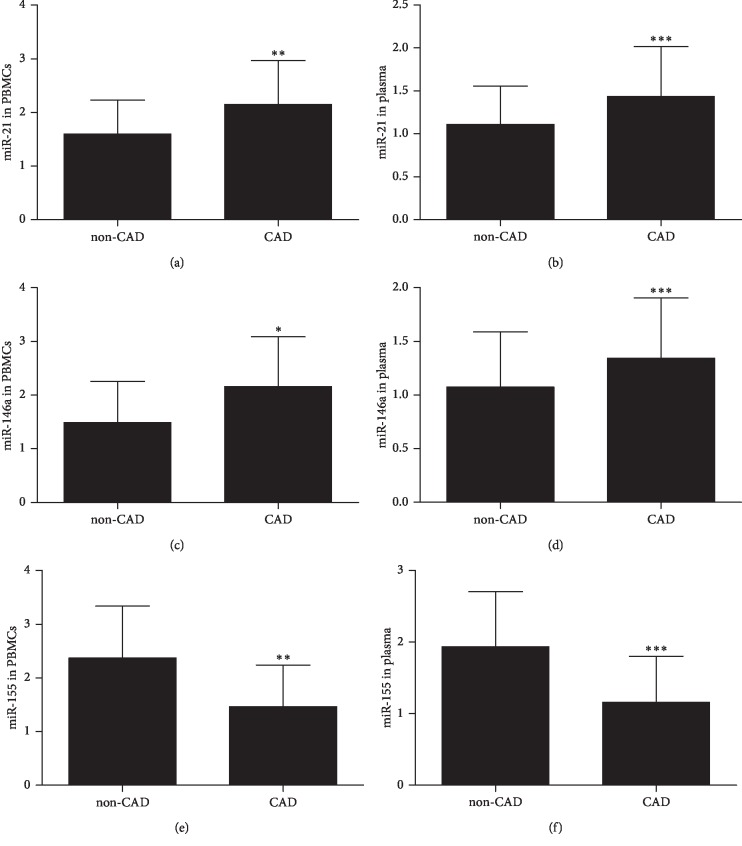
The levels of miR-21, miR-146a, and miR-155 in PBMCs (a, c, e) and in plasma (b, d, f) between CAD and non-CAD groups. miR-21, miR-146a, and miR-155 levels were measured with quantitative real-time PCR. Data are shown as mean ± SD. ^*∗*^*P* < 0.05 versus non-CAD groups; ^*∗∗*^*P* < 0.01 versus non-CAD groups; ^*∗∗∗*^*P* < 0.001 versus non-CAD groups. CAD, coronary artery disease; PBMC, peripheral blood mononuclear cell.

On the basis of angiography, the patients with coronary stenosis of ≥50% were categorized as CAD group (*n* = 203), and those with coronary stenosis of <50% were categorized as non-CAD group (*n* = 107). The expression of miR-21 in PBMCs of CAD group was significantly higher than that in PBMCs of the non-CAD group (2.15 ± 0.06 vs. 1.60 ± 0.06; *P*=0.0041) (Figure 1(a)). The expression of miR-146a in PBMCs of CAD group was significantly higher than that in PBMCs of the non-CAD group (2.16 ± 0.07 vs. 1.49 ± 0.07; *P*=0.0317) (Figure 1(c)). Although the expression of miR-155 in PBMCs of CAD group was significantly lower than that in PBMCs of the non-CAD group (1.46 ± 0.05 vs. 2.37 ± 0.09; *P*=0.0096) (Figure 1(e)). In plasma, expression patterns of miR-21, miR-146a, and miR-155 were similar as that of PBMCs. The expression of miR-21, miR-146a, and miR-155 in plasma of CAD group was significantly different from that in plasma of the non-CAD group (1.43 ± 0.04 vs. 1.11 ± 0.04, 1.35 ± 0.04 vs. 1.07 ± 0.05, 1.16 ± 0.05 vs. 1.94 ± 0.08; *P* < 0.001) (Figure 1(b), 1(d), and 1(f)).

### 3.3. The Altered Expression of miR-21, miR-146a, and miR-155 in Patients with CAD

**Figure 2:**
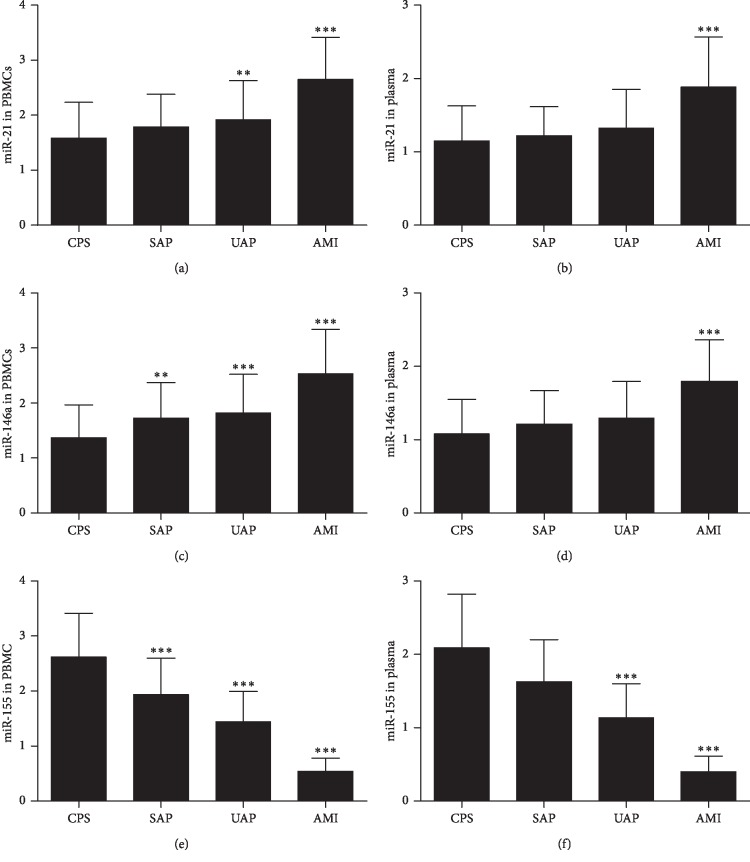
The levels of miR-21, miR-146a, and miR-155 in PBMCs (a, c, e) and in plasma (b, d, f) from patients with SA, UA, and AMI compared with those with CPS. Data are shown as mean ± SD. ^*∗∗*^*P* < 0.01 versus CPS; ^*∗∗∗*^*P* < 0.001 versus CPS. CPS, chest pain syndrome; SAP, stable angina pectoris; UAP, unstable angina pectoris; AMI, acute myocardial infarction; PBMCs, peripheral blood mononuclear cells.

The miR-21 expression in PBMCs was higher in the UAP group (*P*=0.008) and AMI group (*P*=0.001) than in the CPS group, and the miR-21 expression in PBMCs was higher in the AMI group than in the UAP group (*P*=0.033) and SAP group (*P*=0.010), but the difference between the UAP group and the SAP group is not significant (Figure 2(a)). The miR-146a expression in PBMCs was higher in thSAP (*P*=0.010), UAP (*P*=0.000), and AMI groups (*P*=0.000) than in the CPS group, and the miR-146a expression in PBMCs was higher in AMI group than in the UAP group (*P*=0.042) and SAP group (*P*=0.015), but the difference between the UAP group and the SAP group is not significant (Figure 2(c)). Although the level of miR-155 in PBMCs was lower in the SAP group (*P*=0.000), UAP group (*P*=0.000), and AMI group (*P*=0.000) than in the CPS group, and the miR-155 expression in PBMCs was lower in AMI group than in the UAP group (*P*=0.001) and SAP group (*P*=0.000), and the difference between the UAP group and the SAP group is significant (Figure 2(e)). In plasma, expression patterns of miR-21, miR-146a, and miR-155 were similar as in PBMCs. The level of miR-21 and miR-146a in plasma was higher in the AMI group than in the CPS, SAP, and UAP groups, although no statistically significant differences were observed between the UAP group and SAP group (Figure 2(b) and 2(d)). The level of miR-155 in plasma was lower in the UAP and AMI groups than in the CPS group, and the miR-155 expression in plasma was lower in the AMI group than in the UA and SAP groups, although no statistically significant differences were observed between patients in the SAP group and those in the UAP group (Figure 2(f)).

### 3.4. The Relationship between the Expression of miR-21, miR-146a, and miR-155 and Plaque Stability

The relationship between the expression of miR-21, miR-146a, and miR-155 in plasma and in PBMCs and plaque stability was analyzed. IVUS was used to evaluate the plaque stability; 100 patients with CAD underwent IVUS and were divided into 2 groups: stable plaque group (*n* = 48) and vulnerable plaque group (*n* = 52). This analysis showed that the expression of miR-21 and miR-146a in PBMCs was higher in the vulnerable plaque group than in the stable plaque group (2.72 ± 0.68 vs. 1.58 ± 0.43; *P*=0.032; 2.86 ± 0.75 vs. 1.44 ± 0.38; *P*=0.028), whereas the expression of miR-155 in PBMCs was lower in the vulnerable plaque group than in the stable plaque group (2.79 ± 1.05 vs. 3.15 ± 1.23; *P*=0.042). In plasma, the expression of miR-21 and miR-146a was higher in the vulnerable plaque group than in the stable plaque group (2.13 ± 0.54 vs. 1.06 ± 0.25; *P*=0.029; 1.88 ± 0.48 vs. 1.12 ± 0.21; *P*=0.034), whereas the expression of miR-155 was lower in the vulnerable plaque group than in the stable plaque group (2.12 ± 0.89 vs. 2.88 ± 1.02; *P*=0.039). Because there is a difference in IL6, IL8, and hs-CRP among AMI group, UAP group, and CPS group, we also analyzed the level of IL-6, IL-8, and hs-CRP between stable plaque group and vulnerable plaque group. This analysis showed that there was no significant difference in the level of IL-6, IL-8, and hs-CRP between the stable plaque group and the vulnerable plaque group (Table 2).

Hundred patients with CAD underwent IVUS and divided them into 3 groups: calcified plaque group (*n* = 30), fibrous plaque group (*n* = 32), and soft plaque group (*n* = 38). The expression of miR-21 and miR-146a in PBMCs was higher in the soft plaque group than in the calcified plaque group (2.88 ± 0.84 vs. 1.44 ± 0.38; *P*=0.025; 3.36 ± 0.91 vs. 1.40 ± 0.31; *P*=0.016), whereas the expression of miR-155 in PBMCs was lower in the soft plaque group than in the calcified plaque group (1.53 ± 0.78 vs. 3.52 ± 1.36; *P*=0.015). In plasma, the expression of miR-21 and miR-146a was higher in the soft plaque group than in the calcified plaque group (2.66 ± 0.73 vs. 0.98 ± 0.15; *P*=0.028; 2.23 ± 0.65 vs. 1.18 ± 0.18; *P*=0.039), whereas the expression of miR-155 was lower in the soft plaque group than in the calcified plaque group (1.22 ± 0.46 vs. 3.02 ± 1.24; *P*=0.012). In addition, the expression of miR-146a in PBMCs and plasma was higher in the soft plaque group than in the fibrous plaque group (3.36 ± 0.91 vs. 2.86 ± 0.75; *P*=0.043; 2.23 ± 0.65 vs. 1.86 ± 0.48; *P*=0.045). However, there were no significant differences in level of IL-6, IL-8, and hs-CRP in different groups (Table 3).

## 4. Discussion

Inflammation contribute to the initiation and progression of atherosclerosis. Recent studies have indicated that miR-21, miR-146a, and miR-155 were involved in inflammatory diseases. To evaluate whether miR-21, miR-146a, and miR-155 is involved in the progress of atherosclerosis, we studied the relative expression of miR-21, miR-146a, and miR-155 in both plasma and PBMCs of patients with CAD and non-CAD patients. We found that the miR-21 and miR-146a expression in PBMCs and plasma was significantly higher in CAD patients than in those in the non-CAD group. However, the miR-155 expression in PBMCs and plasma was significantly lower in patients with CAD than in those in the non-CAD group. These results illustrated that miR-21, miR-146a, and miR-155 were closely related to coronary artery disease, but whether they are related to the severity of coronary artery disease is not known. We divided patient with chest pain into 4 groups: AMI, UAP, SAP, and CPS group. There were no significant differences in gender, age, and risk factors (hypertension, diabetes, hyperlipidemia, tobacco) among patients with CPS, SAP, UAP, and AMI. However, the levels of hs-CRP, IL-6, and IL-8 were significantly increased in the UAP and AMI groups compared with the CPS groups. These results were consistent with Tayefi et al. [17] and Tajfard et al. [18]. They found that hs-CRP was strongly associated with coronary heart disease (CHD), and the combination of MCP-1 and IL-6 can predict the presence of coronary artery disease and mortality. Our results also demonstrated that miR-21 was upregulated in PBMCs in patients with UAP and AMI, and miR-146a was upregulated in PBMCs in patients with SAP, UAP, and AMI, whereas miR-155 was downregulated in PBMCs in patients with SAP, UAP, and AMI. Additionally, the expression patterns of miR-21, miR-146a, and miR-155 in plasma were similar with PBMCs. These results were consistent with our previous study [12]and those of Yao et al. [11]. These results demonstrated that miR-21, miR-146a, and miR-155 were related to the severity of coronary artery disease. MiR-21 and miR-146a increased with the severity of coronary artery disease, whereas miR-155 decreased with the severity of coronary artery disease, which suggested a harmful effect of miR-21 and miR-146a and a protective role of miR-155 in atherosclerosis.

Studies have shown that the occurrence and prognosis of myocardial infarction depends more on the composition and stability of atherosclerotic plaques than on the degree of coronary artery stenosis. More than 68.5% of myocardial infarction is caused by vulnerable plaque [19]. But the relationship between miR-21, miR-146a, and miR-155 and plaque stability is unknown. IVUS was introduced to evaluate the plaque stability of coronary stenotic lesions. The result showed that the expression of miR-21 and miR-146a in PBMCs and plasma were higher in vulnerable plaque group than that in the stable plaque group (*P* < 0.05), whereas the expression of miR-155 in PBMCs and plasma were lower in the vulnerable plaque group than that in the stable plaque group (*P* < 0.05). We also found that the expression of miR-21 and miR-146a in PBMCs and plasma were higher in the soft plaque group compared with calcified plaque group, whereas the expression of miR-155 in PBMCs and plasma were lower in the soft plaque group than in the calcified plaque group. In addition, the expression of miR-146a in PBMCs and plasma were higher in the soft plaque group than in the fibrous plaque group. These results showed that miR-21 and miR-146a are positively related to plaque stability, whereas miR-155 is negatively related to plaque stability. Because there was a difference in IL6, IL8, and hs-CRP among AMI, UAP, and CPS groups, we analyzed the level of IL-6, IL-8, and hs-CRP between stable plaque group and vulnerable plaque group. This analysis showed that there was no significant difference in the level of IL-6, IL-8, and hs-CRP between stable plaque group and vulnerable plaque group. We also found that there were no significant differences in the level of IL-6, IL-8, and hs-CRP in different groups (calcified plaque group, fibrous plaque group, and soft plaque group). These results showed that miR-21, miR-146a, and miR-155 were more likely to be biomarkers of plaque instability compared with some inflammatory factors such as IL-6, IL-8, and hs-CRP.

MiR-21 is considered as an inflammation-related microRNAs and a new biomarker of inflammation [20]. Sygitowicz et al. [21] found that circulating miR-21 overexpression was seen in all patients with heart failure, independent of heart failure severity. Sheane et al. [22] found that miR-21 is increased in the aorta in atherosclerosis, which was consistent with our study. It showed close relationship between miR-21 and coronary artery disease. But Eryılmaz et al. [23] found that miR-21 expression levels in STEMI patients showed no significant differences compared with control patients, which is not consistent with our study. That may be due to the deference of sample size because they included only 12 patients. Little is known about the relationship between miR-21 and plaque stability. In this study, we found that miR-21 level was significantly higher in the vulnerable plaque group than in the stable plaque group, which support a harmful role of miR-21 in atherosclerosis. But Cheng et al. [24] suggested that miR-21 protects against the H(2)O(2)-induced injury on cardiac myocytes via its target gene PDCD4, which indicated that miR-21 might play an protective role in heart diseases. The discrepancy may be due to the difference of the cell model.

MiR-146a is also related to inflammation. Taganov et al. [25] showed that miR-146a increased in LPS-stimulated monocytes, TNF*α*, and IL1*β* could also upregulate the expression of miR-146a through NFκB-dependent pathway. In this study, we found that the miR-146a expression in PBMCs and plasma was significantly higher in CAD patients, which was consistent with Yao et al. [11]. In this study, we found that miR-146a level was significantly higher in the vulnerable plaque group than in the stable plaque group, which support a harmful role of miR-146a in atherosclerosis. But Zeng et al. [26] found that miR-146a overexpression could negatively regulate tumor necrosis factor receptor–associated factor 6 (TRAF6) and interleukin-1 receptor–associated kinase-1 (IRAK1) and finally inhibit inflammation in acute lung injury rat model. Gao et al. [27] found that LPS could stimulate the expression of miR-146a in cardiomyocytes and macrophages, and the overexpression of miR-146a could reduce the inflammatory response of cardiomyocytes induced by LPS. These studies reveal the negative feedback effect of miR-146a in inflammatory pathway, which means that inflammation can stimulate the expression of miR-146a in many kinds of cells, and miR-146a can also inhibit inflammation in turn, which indicated that miR-146a might play an protective role in heart diseases. The discrepancy may be due to the difference of the cell model.

MiR-155 have been shown to regulate inflammatory processes. However, we found that miR-155 was downregulated in patients with ACS, which was consistent with our previous study [12] and the study done by Yao et al. [11]. Recently, the associations of miR-155 and coronary atherosclerosis were investigated in several studies. Qiu et al. [28] found that CAD patients had higher miR-155 level in comparison to controls, and the miR-155 content significantly increased following an increasing Gensini score, which showed serum miR-155 may serve as a novel biomarker for evaluating severity of CAD. In addition, our previous study [29]suggested that miR-155 regulates the angiotensin II–induced vascular smooth muscle cell proliferation, which may play an important role in AS. Donners et al. [30] reported anti-inflammatory and atheroprotective effects of miR-155 on atherosclerosis in vivo hyperlipidemic mice. They found that miR-155 deficiency promoted atherosclerotic plaque development and decreased plaque stability. Zhu et al. [31] also found that miR-155 can prevent the development and progression of atherosclerosis by targeting MAP3K10. In this study, we introduced the IVUS to assess the plaque stability of coronary stenotic lesions. Our study is the first one to show that miR-155 is negatively associated with the plaque stability of coronary stenotic lesions. These findings supports a significant protective role of miR-155 on atherosclerosis and plaque stability. Reversely, our previous study [32] revealed that miR-155 overexpression significantly promotes inflammatory cytokine and chemokine production and hence the atherosclerosis progression. Similarly, Nazari-Jahantigh et al. [33] found that microRNA-155 promotes atherosclerosis by repressing Bcl6 in macrophages. These findings supported a harmful effect of miR-155 on atherosclerosis and plaque stability. The discrepancy in the studies may be due to difference in the study models and also the analysis method of plaque stability.

In addition, some studies have shown that gender and sex difference in coronary plaque composition was present. For example, Pundziute et al [34] and Blaha et al [35] found that more calcified lesions were observed in men than in women. So, theoretically there are some gender differences in expression of miR-21, miR-146a, and miR-155, but in this study, we did not find any gender difference in the expression of miR-21, miR-146a, and miR-155. Maybe, it is because of the small sample size.

## 5. Conclusions

In summary, our study found that miR-21 and miR-146a were evidently upregulated in patients with ACS, whereas miR-155 was evidently downregulated in patients with ACS. Moreover, this study is the first to show that miR-21 and miR-146a were associated with plaque stability, whereas miR-155 expression is inversely associated with plaque stability, supporting a harmful effect of miR-21 and miR-146a and a protective role of miR-155 in atherosclerosis.

## 6. Limitations

There are some limitations of this study: first one is we had limited number of subjects in this study. Second, we were not able to use more established methods to evaluate the plaque instability, such as Virtual Histology Intravascular Ultrasound and OCT. The third one is we had given the limited classification of the plaques and not been able to classify plaques as mixed plaque, thrombus, dissection due to UA and AMI scenarios. Finally, the mechanism of action is not understood well yet. Hence, it necessitates further studies in future to clarify the mechanism of action of miR-21, miR-146a, and miR-155 in plaque stability of coronary stenotic lesions.

## Figures and Tables

**Table 1:** Clinical characteristics of patients.

Characteristics	CPS	SAP	UAP	AMI
Total population	83	88	97	42
Age (mean ± s.d.)	60 ± 8	62 ± 8	63 ± 7	62 ± 7
Sex (male/female)	50/33	52/36	65/32	28/14
Risk factors				
Hypertension	38 (46%)	40 (45%)	40 (41%)	14 (33%)
Diabetes	19 (23%)	32 (36%)	39 (40%)	13 (31%)
Hyperlipidemia	30 (36%)	36 (41%)	42 (43%)	10 (24%)
Tobacco	29 (35%)	31 (35%)	37 (38%)	16 (38%)
hs-CRP (mg/L)	1.15 ± 0.39	1.76 ± 0.59	2.57 ± 0.90^*∗*^	3.78 ± 0.70^*∗*&#^
IL-6 (ng/L)	2.32 ± 1.07	4.01 ± 0.98	8.99 ± 2.98^*∗*&^	11.95 ± 4.20^*∗*&#^
IL-8 (ng/L)	16.40 ± 6.13	72.28 ± 19.59^*∗*^	85.94 ± 13.00^*∗*^	104.75 ± 22.16^*∗*&#^

CPS, chest pain syndrome; SAP, stable angina pectoris; UAP, unstable angina pectoris; AMI, acute myocardial infarction; hs-CRP, high-sensitivity C-reactive protein. ^*∗*^*P* < 0.01 versus CPS; ^&^*P* < 0.05 versus SAP; ^#^*P* < 0.05 versus UAP.

**Table 2:** The PBMCs and plasma levels of miR-21, miR-146a, and miR-155 in stable plaque group and vulnerable plaque group.

Goupe	*n*	miR-21 in PBMCs	miR-21 in plasma	miR-146a in PBMCs	miR-146a in plasma	miR-155 in PBMCs	miR-155 in plasma	IL-6	Il-8	hs-CRP
Stable plaque	48	1.58 ± 0.43	1.06 ± 0.25	1.44 ± 0.38	1.12 ± 0.21	3.15 ± 1.23	2.88 ± 1.02	5.58 ± 2.68	73.43 ± 23.26	2.23 ± 0.96
Vulnerable plaque	52	2.72 ± 0.68	2.13 ± 0.54	2.86 ± 0.75	1.88 ± 0.48	2.79 ± 1.05	2.12 ± 0.89	6.97 ± 2.93	79.22 ± 17.47	2.30 ± 0.90
*P* value		0.032	0.029	0.028	0.034	0.042	0.039	0.548	0.057	0.665

**Table 3:** The PBMCs and plasma levels of miR-21, miR-146a, and miR-155 in calcified plaque group, fibrous plaque group, and soft plaque group.

Goupe	*n*	miR-21 in PBMCs	miR-21 in plasma	miR-146a in PBMCs	miR-146a in plasma	miR-155 in PBMCs	miR-155 in plasma	IL-6	Il-8	hs-CRP
Calcified plaque	30	1.44 ± 0.38	0.98 ± 0.15	1.40 ± 0.31	1.18 ± 0.18	3.52 ± 1.36	3.02 ± 1.24	5.60 ± 2.24	74.94 ± 24.94	2.25 ± 0.89
Fibrous plaque	32	1.72 ± 0.58	1.58 ± 0.36	2.86 ± 0.75^*∗*^	1.86 ± 0.48^*∗*^	2.79 ± 1.05	2.12 ± 0.89	5.95 ± 3.26	75.55 ± 22.22	1.98 ± 0.93
Soft plaque	38	2.88 ± 0.84^*∗*^	2.66 ± 0.73^*∗*^	3.36 ± 0.91^*∗*#^	2.23 ± 0.65^*∗*#^	1.53 ± 0.78^*∗*^	1.22 ± 0.46^*∗*^	7.14 ± 2.86	80.74 ± 13.89	2.52 ± 0.91

^*∗*^ *P* < 0.05 versus calcified plaque group; ^#^*P* < 0.05 versus fibrous plaque group.

## Data Availability

All the data used in the analysis are presented within the manuscript.

## Supplementary Materials

Supplementary Materials The results of CAG (CAD, non-CAD) in SAP group, UAP group, AMI group.
